# Supplementary material for: Interactions between metabolism and growth can determine the co-existence of Staphylococcus aureus and Pseudomonas aeruginosa
Source: eLife. 2023 Apr 20;12:e83664. doi: 10.7554/eLife.83664 (PMC10174691; doi:10.7554/eLife.83664)
Supplement: Supplementary file 1. — (a) P values for data presented in Figure 1. n represents the number of biological replicates. To calculate absolute growth, all biological replicates from maximum growth rate and [ATP] were used. Thus, the value of n for absolute growth represents the smallest number of biological replicates included in the calculation. [file elife-83664-supp1.docx]

**Supplementary file 1a**

| **Figure panel** | **Carbon source** | ***n*** | **P value** |
| --- | --- | --- | --- |
| Figure 1B  (maximum growth rate) | Acetate | 3 | < 0.001 |
|  | α-ketoglutarate | 4 | 0.370 |
|  | Galactose | 3 | <0.001 |
|  | Glucose | 3 | <0.001 |
|  | Glycerol | 3 | 0.0495 |
|  | Lactic acid | 3 | 0.088 |
|  | Lactose | 3 | 0.027 |
|  | Mannose | 3 | 0.011 |
|  | Pyruvate | 3 | <0.001 |
|  | Ribose | 3 | 0.002 |
|  | Sorbitol | 3 | 0.004 |
|  | Succinate | 4^*^ | 0.694 |
|  | Sucrose | 3 | <0.001 |
| Figure 1C  (concentration of ATP) | Acetate | 5 | 0.010 |
|  | α-ketoglutarate | 4^*^ | 0.03 |
|  | Galactose | 5 | 0.061 |
|  | Glucose | 5 | 0.005 |
|  | Glycerol | 5 | 0.008 |
|  | Lactic acid | 5 | <0.001 |
|  | Lactose | 5 | 0.168 |
|  | Mannose | 5 | <0.001 |
|  | Pyruvate | 5 | 0.048 |
|  | Ribose | 5 | 0.003 |
|  | Sorbitol | 5 | 0.056 |
|  | Succinate | 4^*^ | 0.425 |
|  | Sucrose | 5 | 0.009 |
| Figure 1D  (absolute growth) | Acetate | 3 | 0.002 |
|  | α-ketoglutarate | 4 | 0.025 |
|  | Galactose | 3 | 0.133 |
|  | Glucose | 3 | 0.073 |
|  | Glycerol | 3 | 0.987 |
|  | Lactic acid | 3 | 0.927 |
|  | Lactose | 3 | 0.576 |
|  | Mannose | 3 | <0.001 |
|  | Pyruvate | 3 | 0.011 |
|  | Ribose | 3 | 0.046 |
|  | Sorbitol | 3 | 0.085 |
|  | Succinate | 4 | 0.283 |
|  | Sucrose | 3 | 0.013 |

*n for *P. aeruginosa* = 5; for *S. aureus* = 4
