# Supplementary material for: Interactions between metabolism and growth can determine the co-existence of Staphylococcus aureus and Pseudomonas aeruginosa
Source: eLife. 2023 Apr 20;12:e83664. doi: 10.7554/eLife.83664 (PMC10174691; doi:10.7554/eLife.83664)
Supplement: Supplementary file 2. — (a) Base parameter set used in our mathematical model (Equations 1–3). (b) P values for data presented in Figure 2. n represents the number of biological replicates. Shapiro-Wilk for data sets in Figure 2A and B, P<0.0001. In Figure 2B and a Dunn test for joint rank was used to compare final density ratios. The final density ratio of the wildtype P. aeruginosa strain co-cultured with S. aureus was used as the control. TSB supplemented with glucose was used for these experiments. [file elife-83664-supp2.docx]

**Supplementary file 2a**

| **Parameter (symbol)** | **Value, units** | **Reference** |
| --- | --- | --- |
| Synthesis rate of virulence factors (*k_p_*) | 1 μM/hr | (17, 58, 62) |
| Decay rate of virulence factors (*d_v_*) | 0.1/hr | (63, 64) |
| Death rate of bacteria (*d_c_*) | 0.05/hr | (65) |
| Growth rate of *S. aureus* (*μ_s_*) | 0.4/hr | Figure 1B |
| Growth rate of *P. aeruginosa* (*μ_p_*) | 0.32/hr | Figure 1B |
| Metabolism of *S. aureus* (*ε_s_*) | 1 mmol/g/hr | (59-61) |
| Metabolism of *P. aeruginosa* (*ε_p_*) | 0.8 mmol/g/hr | (59-61) |
| Half maximal production rate of virulence factors (*K*) | 0.1 | Estimated, see Methods |
| Spatial distribution (*δ* ) | 0.1-1 | Estimated, see Methods |

**Supplementary file 2b**

| **Figure panel** | **Carbon source (*P. aeruginosa* strain)** | ***n*** | **P value** | |
| --- | --- | --- | --- | --- |
|  | | | Mann-Whitney (for bacterial densities) | |
| Figure 2A | α-ketoglutarate (PA14) | 4 | 0.0209 | |
|  | Glucose (PA14) | 5 | 0.009 | |
|  | Lactic acid (PA14) | 4 | 0.0606 | |
|  | Pyruvate (PA14) | 3 | 0.0463 | |
|  | Ribose (PA14) | 4 | 0.0283 | |
|  | Succinate (PA14) | 5 | 0.009 | |
|  | Sucrose (PA14) | 4 | 0.0833 | |
|  | | | Kruskal- Wallis | Dunn with control (wildtype) for joint ranks. |
| Figure 2B | Glucose (Δ*pqsL*) | 5 | 0.0051 | 0.0309 |
|  | Glucose (Δ*pvdA*/Δ*pchE*) | 5 |  | 0.163 |
|  | Glucose (Δ*pqsL*/Δ*pchE*/Δ*pvdA*) | 5 |  | 0.0023 |
|  | | | ANOVA | (Dunnett’s with control (wildtype) for joint ranks.) |
| Figure 2 – figure supplement 1 | α-ketoglutarate (Δ*pqsL*) | 3 | 0.774 | 0.6073 |
|  | α-ketoglutarate (Δ*pvdA*/Δ*pchE*) | 3 |  | 0.86 |
|  | α-ketoglutarate (Δ*pqsL*/Δ*pchE*/Δ*pvdA*) | 3 |  | 0.9746 |
|  | Glucose (Δ*pqsL*) | 9 | 0.4990 | 0.324 |
|  | Glucose (Δ*pvdA*/Δ*pchE*) | 9 |  | 0.242 |
|  | Glucose (Δ*pqsL*/Δ*pchE*/Δ*pvdA*) | 6 |  | 0.316 |
|  | Lactic acid (Δ*pqsL*) | 4 | 0.0167 | 0.1911 |
|  | Lactic acid (Δ*pvdA*/Δ*pchE*) | 4 |  | 0.9953 |
|  | Lactic acid (Δ*pqsL*/Δ*pchE*/Δ*pvdA*) | 4 |  | 0.2164 |
|  | Pyruvate (Δ*pqsL*) | 4 | 0.2635 | 1 |
|  | Pyruvate (Δ*pvdA*/Δ*pchE*) | 4 |  | 0.483 |
|  | Pyruvate (Δ*pqsL*/Δ*pchE*/Δ*pvdA*) | 4 |  | 0.2745 |
|  | Ribose (Δ*pqsL*) | 4 | 0.2765 | 0.7988 |
|  | Ribose (Δ*pvdA*/Δ*pchE*) | 4 |  | 0.3952 |
|  | Ribose(Δ*pqsL*/Δ*pchE*/Δ*pvdA*) | 4 |  | 0.1656 |
|  | Succinate (Δ*pqsL*) | 4 | 0.0814 | 0.8478 |
|  | Succinate (Δ*pvdA*/Δ*pchE*) | 3 |  | 0.4154 |
|  | Succinate (Δ*pqsL*/Δ*pchE*/Δ*pvdA*) | 4 |  | 0.0429 |
|  | Sucrose (Δ*pqsL*) | 3 | 0.6387 | 0.996 |
|  | Sucrose (Δ*pvdA*/Δ*pchE*) | 4 |  | 0.8015 |
|  | Sucrose (Δ*pqsL*/Δ*pchE*/Δ*pvdA*) | 4 |  | 0.5173 |
