# Supplementary material for: Interactions between metabolism and growth can determine the co-existence of Staphylococcus aureus and Pseudomonas aeruginosa
Source: eLife. 2023 Apr 20;12:e83664. doi: 10.7554/eLife.83664 (PMC10174691; doi:10.7554/eLife.83664)
Supplement: Supplementary file 3. — (a) P values for data presented in Figure 2—figure supplement 1, panel H. Shapiro-Wilk for final density ratios, P=0.0004; for final bacterial densities, P<0.0001. n represents the number of biological replicates. [file elife-83664-supp3.docx]

**Supplementary file 3a**

| **Carbon source** | **Kruskal-Wallis** (for final density ratios). | ***n*** | **P value** (Mann- Whitney, final bacterial densities) |
| --- | --- | --- | --- |
| Sucrose | 0.013 | 3 | 0.0495 |
| Pyruvate |  | 3 | 0.0495 |
| Glucose |  | 6 | 0.0039 |
