# Supplementary material for: Interactions between metabolism and growth can determine the co-existence of Staphylococcus aureus and Pseudomonas aeruginosa
Source: eLife. 2023 Apr 20;12:e83664. doi: 10.7554/eLife.83664 (PMC10174691; doi:10.7554/eLife.83664)
Supplement: Supplementary file 4. — (a) P values for data presented in Figure 3. n represents the number of biological replicates. To calculate absolute growth, all biological replicates from maximum growth rate and ATP were used. Thus, the value of n for absolute growth represents the smallest number of biological replicates included in the calculation. Two-tailed t-tests were used for [ATP], growth rate and absolute growth measurements. A Mann-Whitney (Shapiro-Wilk, P<0.0001) was used to assess differences in the final density of P. aeruginosa and S. aureus (Figure 3E and J). [file elife-83664-supp4.docx]

**Supplementary file 4a**

| **Figure panel** | **Carbon source** | ***n* (*P. aeruginosa*/*S. aureus*)** | **P value** |
| --- | --- | --- | --- |
| Figure 3A (maximum growth rate, AMM) | α-ketoglutarate | 10/9 | < 0.0001 |
|  | Glucose | 10/14 | 0.28 |
|  | Lactic acid | 11/16 | 0.0009 |
|  | Pyruvate | 10/9 | 0.0007 |
|  | Ribose | 8/12 | 0.09 |
|  | Succinate | 8/12 | 0.0033 |
|  | Sucrose | 10/10 | 0.0019 |
| Figure 3B ([ATP], AMM) | α-ketoglutarate | 6/6 | 0.002 |
|  | Glucose | 6/6 | 0.091 |
|  | Lactic acid | 6/6 | 0.15 |
|  | Pyruvate | 6/6 | 0.011 |
|  | Ribose | 6/6 | 0.001 |
|  | Succinate | 6/6 | 0.0005 |
|  | Sucrose | 6/6 | 0.006 |
| Figure 3C (absolute growth, AMM) | α-ketoglutarate | 6/6 | 0.62 |
|  | Glucose | 6/6 | 0.03 |
|  | Lactic acid | 6/6 | 0.18 |
|  | Pyruvate | 6/6 | 0.78 |
|  | Ribose | 6/6 | 0.011 |
|  | Succinate | 6/6 | 0.089 |
|  | Sucrose | 6/6 | 0.006 |
| Figure 3F (maximum growth rate, SCFM) | Glucose | 7/4 | 0.035 |
|  | Lactic acid | 9/6 | <0.0001 |
|  | Pyruvate | 7/5 | 0.61 |
|  | Ribose | 7/6 | 0.166 |
|  | Sucrose | 8/8 | 0.59 |
| Figure 3G ([ATP], SCFM) | Glucose | 5/5 | 0.22 |
|  | Lactic acid | 5/5 | 0.037 |
|  | Pyruvate | 5/5 | 0.092 |
|  | Ribose | 5/5 | 0.14 |
|  | Sucrose | 5/5 | 0.04 |
| Figure 3H (absolute growth, SCFM) | Glucose | 5/5 | 0.99 |
|  | Lactic acid | 5/5 | 0.25 |
|  | Pyruvate | 5/5 | 0.14 |
|  | Ribose | 5/5 | 0.0132 |
|  | Sucrose | 5/5 | 0.0135 |
| Figure 3E (final density ratio vs. absolute growth ratio) | α-ketoglutarate | 5 | 0.009 |
|  | Glucose | 7 | 0.0017 |
|  | Lactic acid | 5 | 0.009 |
|  | Pyruvate | 7 | 0.0017 |
|  | Ribose | 5 | 0.009 |
|  | Succinate | 14 | <0.0001 |
|  | Sucrose | 8 | 0.3431 |
| Figure 3J (final density vs. absolute growth ratio) | Glucose | 8 | 0.0008 |
|  | Lactic acid | 16 | <0.0001 |
|  | Pyruvate | 6 | 0.0039 |
|  | Ribose | 16 | <0.001 |
|  | Sucrose | 12 | 0.0001 |
