# Supplementary material for: Interactions between metabolism and growth can determine the co-existence of Staphylococcus aureus and Pseudomonas aeruginosa
Source: eLife. 2023 Apr 20;12:e83664. doi: 10.7554/eLife.83664 (PMC10174691; doi:10.7554/eLife.83664)
Supplement: Supplementary file 5. — Shapiro-Wilk, P≤0.0001 for all carbon sources. n represents the number of biological replicates. [file elife-83664-supp5.docx]

**Supplementary file 5a**

| **Carbon source** | **Kruskal-Wallis**  (for final density ratios) | ***n*** | **Initial % of *P. aeruginosa*** | **P value**  (Mann-Whitney, for bacterial densities) |
| --- | --- | --- | --- | --- |
| Glucose | 0.0009 | 5 | 60 | 0.0283 |
|  |  | 5 | 50 | 0.009 |
|  |  | 6 | 40 | 0.5653 |
|  |  | 6 | 30 | 0.2492 |
|  |  | 6 | 20 | 0.0088 |
| Lactic acid | 0.0029 | 8 | 60 | 0.0357 |
|  |  | 4 | 50 | 0.0433 |
|  |  | 5 | 40 | 0.009 |
|  |  | 5 | 30 | 0.0472 |
|  |  | 4 | 20 | 0.6631 |
|  |  | 4 | 10 | 0.0209 |
| Pyruvate | < 0.0001 | 7 | 60 | 0.0088 |
|  |  | 3 | 50 | 0.0202 |
|  |  | 4 | 40 | 0.0209 |
|  |  | 6 | 30 | 0.025 |
|  |  | 4 | 20 | 0.0209 |
|  |  | 9 | 10 | 0.0023 |
|  |  | 4 | 1 | 0.0202 |
| Ribose | < 0.0001 | 5 | 60 | 0.0112 |
|  |  | 5 | 50 | 0.0367 |
|  |  | 4 | 40 | 0.0304 |
|  |  | 5 | 30 | 0.0367 |
|  |  | 5 | 20 | 0.1745 |
|  |  | 9 | 10 | 0.757 |
|  |  | 5 | 1 | 0.009 |
| Sucrose | < 0.0001 | 6 | 60 | 0.0374 |
|  |  | 4 | 50 | 0.0833 |
|  |  | 5 | 40 | 0.6015 |
|  |  | 6 | 30 | 0.025 |
|  |  | 3 | 20 | 0.0463 |
