# Supplementary material for: Interactions between metabolism and growth can determine the co-existence of Staphylococcus aureus and Pseudomonas aeruginosa
Source: eLife. 2023 Apr 20;12:e83664. doi: 10.7554/eLife.83664 (PMC10174691; doi:10.7554/eLife.83664)
Supplement: Supplementary file 6. — (a) P values for data presented in Figure 5E, G and F. Shapiro-Wilk for all data sets in this figure; P≤0.019. n represents the number of biological replicates. (b) P value for data presented in Figure 5F, right panel. Shapiro-Wilk for final density ratios, P<0.0001. Shapiro-Wilk for final bacterial densities, P=0.0190. n represents the number of biological replicates. [file elife-83664-supp6.docx]

**Supplementary file 6a**

| **Figure panel** | **Shaking frequency (/hr)** | **Kruskal- Wallis**  (for final density ratio) | ***n*** | **Carbon source** | ***P. aeruginosa* strain** | **P value**  (Mann- Whitney, for bacterial densities) |
| --- | --- | --- | --- | --- | --- | --- |
| Figure 5E | 0 | < 0.0001 | 5 | Glucose | PA14 | 0.009 |
|  | 1 |  | 4 |  |  | 0.2482 |
|  | 3 |  | 4 |  |  | 0.0202 |
|  | 6 |  | 4 |  |  | 0.0202 |
|  | 9 |  | 4 |  |  | 0.0202 |
|  | 12 |  | 6 |  |  | 0.0161 |
|  | 15 |  | 6 |  |  | 0.0039 |
|  | 18 |  | 6 |  |  | 0.0039 |
|  | 20 |  | 5 |  |  | 0.6004 |
|  | continuous |  | 3 |  |  | 0.0495 |
| Figure 5G | 6 | 0.0023 | 4 | Glucose |  | 0.0202 |
|  |  |  | 3 | Lactic acid |  | 0.0495 |
|  |  |  | 5 | Pyruvate |  | 0.0163 |
|  |  |  | 3 | Ribose |  | 0.0495 |
|  |  |  | 6 | Sucrose |  | 0.0039 |
|  | continuous | 0.0366 | 3 | α-ketoglutarate |  | 0.0495 |
|  |  |  | 6 | Glucose |  | 0.0495 |
|  |  |  | 3 | Lactic acid |  | 0.0039 |
|  |  |  | 5 | Pyruvate |  | 0.0495 |
|  |  |  | 6 | Ribose |  | 0.0086 |
|  |  |  | 3 | Sucrose |  | 0.0039 |
| Figure 5H | 6 | 0.014 | 3 | Glucose | Dunn with control (wildtype) for joint ranks. | |
|  |  |  | 5 |  | (Δ*pqsL*) | 0.1134 |
|  |  |  | 4 |  | (Δ*pvdA*/Δ*pchE*) | 0.0834 |
|  |  |  | 5 |  | (Δ*pqsL*/Δ*pchE*/Δ*pvdA*) | 0.0054 |
|  | continuous | 0.0034 | 6 | Glucose | (Δ*pqsL*) | 0.0129 |
|  |  |  | 5 |  | (Δ*pvdA*/Δ*pchE*) | 1 |
|  |  |  | 5 |  | (Δ*pqsL*/Δ*pchE*/Δ*pvdA*) | 0.0109 |

**Supplementary file 6b**

| **Initial percentage of *P. aeruginosa*** | **Kruskal-Wallis**  (for all final density ratios) | ***n*** | **P value** (Mann- Whitney, between final bacterial densities) |
| --- | --- | --- | --- |
| 50 | 0.115 | 4 | 0.0202 |
| 70 |  | 3 | 0.1266 |
| 75 |  | 5 | 0.8273 |
| 80 |  | 3 | 0.8273 |
| 99 |  | 4 | 0.1465 |
