# Supplementary material for: Interactions between metabolism and growth can determine the co-existence of Staphylococcus aureus and Pseudomonas aeruginosa
Source: eLife. 2023 Apr 20;12:e83664. doi: 10.7554/eLife.83664 (PMC10174691; doi:10.7554/eLife.83664)
Supplement: Supplementary file 7. — (a) P values for data presented in Figure 5—figure supplement 3. Shapiro-Wilk for final density ratio and for bacterial densities, P<0.0001. [file elife-83664-supp7.docx]

**Supplementary file 7a**

| **Shaking frequency (/hr)** | **Kruskal-Wallis**  (for final density ratios) | ***n*** | **P value** (Mann-Whitney, between final bacterial densities. |
| --- | --- | --- | --- |
| 0 | 0.0004 | 8 | 0.0008 |
| 6 |  | 5 | 0.009 |
| continuous |  | 6 | 0.0033 |
