# Supplementary material for: Interactions between metabolism and growth can determine the co-existence of Staphylococcus aureus and Pseudomonas aeruginosa
Source: eLife. 2023 Apr 20;12:e83664. doi: 10.7554/eLife.83664 (PMC10174691; doi:10.7554/eLife.83664)
Supplement: Supplementary file 8. — (a) Average residual values for all curve fitting and biological replicates. Curve fitting was used to determine maximum growth rate in Figure 1B (TSB medium). (b) Average residual values for all curve fitting and biological replicates. Curve fitting was used to determine maximum growth rate in Figure 3A (AMM medium). (c) Average residual values for all curve fitting and biological replicates. Curve fitting was used to determine maximum growth rate in Figure 3F (SCFM medium). [file elife-83664-supp8.docx]

**Supplementary file 8a**

| **Bacteria** | **Carbon source** | **Biological replicate** | | | | |
| --- | --- | --- | --- | --- | --- | --- |
|  |  | 1 | 2 | 3 | 4 | 5 |
| *P. aeruginosa* | Acetate | 0.126 | 0.150 | 0.134 | - | - |
|  | α-ketoglutarate | 0.110 | 0.095 | 0.267 | 0.029 | - |
|  | Galactose | 0.147 | 0.176 | 0.174 | - | - |
|  | Glucose | 0.112 | 0.086 | 0.096 | - | - |
|  | Glycerol | 0.132 | 0.141 | 0.155 | - | - |
|  | Lactic acid | 0.142 | 0.133 | 0.120 | - | - |
|  | Lactose | 0.206 | 0.123 | 0.458 | - | - |
|  | Mannose | 0.232 | 0.147 | 0.123 | - | - |
|  | Pyruvate | 0.118 | 0.088 | 0.132 | - | - |
|  | Ribose | 0.172 | 0.168 | 0.188 | - | - |
|  | Sorbitol | 0.109 | 0.162 | 0.160 | - | - |
|  | Succinate | 0.374 | 0.711 | 0.117 | 0.106 | - |
|  | Sucrose | 0.087 | 0.123 | 0.170 | - | - |
| *S. aureus* | Acetate | 0.022 | 0.040 | 0.024 | - | - |
|  | α-ketoglutarate | 0.069 | 0.0124 | 0.064 | 0.0397 | - |
|  | Galactose | 0.300 | 0.291 | 0.262 | - | - |
|  | Glucose | 0.053 | 0.074 | 0.048 | - | - |
|  | Glycerol | 0.461 | 0.035 | 0.054 | - | - |
|  | Lactic acid | 0.432 | 0.087 | 0.062 | - | - |
|  | Lactose | 0.494 | 0.517 | 0.468 | - | - |
|  | Mannose | 0.302 | 0.264 | 0.251 | - | - |
|  | Pyruvate | 0.027 | 0.0403 | 0.024 | - | - |
|  | Ribose | 0.076 | 0.065 | 0.054 | - | - |
|  | Sorbitol | 0.028 | 0.039 | 0.031 | - | - |
|  | Succinate | 0.536 | 0.205 | 0.131 | 0.182 | 0.075 |
|  | Sucrose | 0.128 | 0.168 | 0.131 | - | - |

**Supplementary file 8b**

| **Bacteria** | **Carbon source** | **Biological replicate** | | | | | | | | | | | | | | | |
| --- | --- | --- | --- | --- | --- | --- | --- | --- | --- | --- | --- | --- | --- | --- | --- | --- | --- |
|  |  | 1 | 2 | 3 | 4 | 5 | 6 | 7 | 8 | 9 | 10 | 11 | 12 | 13 | 14 | 15 | 16 |
| *P. aeruginosa* | α-ketoglutarate | 0.071 | 0.099 | 0.084 | 0.079 | 0.133 | 0.285 | 0.123 | 0.210 | 0.153 | 0.274 | - | - | - | - | - | - |
|  | Glucose | 0.062 | 0.195 | 0.239 | 0.186 | 0.167 | 0.228 | 0.121 | 0.088 | 0.215 | 0.222 | - | - | - | - | - | - |
|  | Lactic acid | 0.075 | 0.114 | 0.365 | 0.322 | 0.103 | 0.128 | 0.217 | 0.199 | 0.139 | 0.122 | 0.297 | - | - | - | - | - |
|  | Pyruvate | 0.034 | 0.122 | 0.154 | 0.112 | 0.156 | 0.214 | 0.222 | 0.261 | 0.200 | 0.410 | - | - | - | - | - | - |
|  | Ribose | 0.108 | 0.127 | 0.290 | 0.232 | 0.219 | 0.178 | 0.284 | 0.279 | 0.279 | - | - | - | - | - | - | - |
|  | Succinate | 0.099 | 0.236 | 0.161 | 0.537 | 0.249 | 0.192 | 0.252 | 0.791 | - | - | - | - | - | - | - | - |
|  | Sucrose | 0.132 | 0.178 | 0.178 | 0.172 | 0.253 | 0.151 | 0.185 | 0.240 | 0.428 | 0.324 | - | - | - | - | - | - |
| *S. aureus* | α-ketoglutarate | 0.036 | 0.017 | 0.026 | 0.022 | 0.055 | 0.070 | 0.062 | 0.126 | 0.198 | - | - | - | - | - | - | - |
|  | Glucose | 0.032 | 0.027 | 0.025 | 0.038 | 0.277 | 0.164 | 0.368 | 0.494 | 0.286 | 0.057 | 0.050 | 0.047 | 0.055 | 0.045 | - | - |
|  | Lactic acid | 0.041 | 0.031 | 0.023 | 0.026 | 0.033 | 0.031 | 0.186 | 0.370 | 0.249 | 0.623 | 0.070 | 0.111 | 0.099 | 0.156 | 0.065 | 0.051 |
|  | Pyruvate | 0.014 | 0.016 | 0.029 | 0.011 | 0.014 | 0.008 | 0.087 | 0.095 | 0.028 | - | - | - | - | - | - | - |
|  | Ribose | 0.052 | 0.023 | 0.018 | 0.030 | 0.020 | 0.015 | 0.123 | 0.058 | 0.086 | 0.234 | 0.178 | 0.316 | - | - | - | - |
|  | Succinate | 0.019 | 0.028 | 0.013 | 0.016 | 0.016 | 0.013 | 0.116 | 0.094 | 0.060 | 0.082 | 0.102 | 0.137 | - | - | - | - |
|  | Sucrose | 0.024 | 0.019 | 0.017 | 0.023 | 0.015 | 0.012 | 0.072 | 0.042 | 0.089 | 0.290 | - | - | - | - | - | - |

**Supplementary file 8c**

| **Bacteria** | **Carbon source** | **Biological replicate** | | | | | | | | |
| --- | --- | --- | --- | --- | --- | --- | --- | --- | --- | --- |
|  |  | 1 | 2 | 3 | 4 | 5 | 6 | 7 | 8 | 9 |
| *P. aeruginosa* | Glucose | 0.097 | 0.172 | 0.387 | 0.833 | 0.171 | 0.338 | 0.350 | - | - |
|  | Lactic acid | 0.184 | 0.096 | 0.182 | 0.345 | 0.323 | 0.297 | 0.356 | 0.606 | 0.271 |
|  | Pyruvate | 0.403 | 0.488 | 0.289 | 0.436 | 0.420 | 0.439 | 0.638 | - | - |
|  | Ribose | 0.189 | 0.213 | 0.119 | 0.326 | 0.588 | 0.447 | 0.527 | - | - |
|  | Sucrose | 0.155 | 0.120 | 0.543 | 0.868 | 0.337 | 0.849 | 0.482 | 0.855 | - |
| *S. aureus* | Glucose | 0.518 | 1.533 | 0.153 | 0.142 | - | - | - | - | - |
|  | Lactic acid | 0.683 | 0.604 | 0.746 | 0.603 | 0.563 | 0.174 | - | - | - |
|  | Pyruvate | 0.677 | 0.750 | 0.067 | 0.043 | 0.083 | - | - | - | - |
|  | Ribose | 0.673 | 0.608 | 0.707 | 0.081 | 0.195 | 0.296 | - | - | - |
|  | Sucrose | 0.586 | 0.568 | 0.556 | 0.531 | 0.148 | 0.120 | 0.142 | 0.054 | - |
